# UK consensus recommendations for clinical management of cancer risk for women with germline pathogenic variants in cancer predisposition genes: *RAD51C*, *RAD51D*, *BRIP1* and *PALB2*

**DOI:** 10.1136/jmg-2022-108898

**Published:** 2022-11-21

**Authors:** Helen Hanson, Anjana Kulkarni, Lucy Loong, Grace Kavanaugh, Bethany Torr, Sophie Allen, Munaza Ahmed, Antonis C Antoniou, Ruth Cleaver, Tabib Dabir, D Gareth Evans, Ellen Golightly, Rosalyn Jewell, Kelly Kohut, Ranjit Manchanda, Alex Murray, Jennie Murray, Kai-Ren Ong, Adam N Rosenthal, Emma Roisin Woodward, Diana M Eccles, Clare Turnbull, Marc Tischkowitz, On behalf of Consensus meeting attendees, Fiona Lalloo

**Affiliations:** 1 South West Thames Regional Genetic Services, St George’s University Hospitals NHS Foundation Trust, London, UK; 2 Division of Genetics and Epidemiology, Institute of Cancer Research, Sutton, UK; 3 Department of Clinical Genetics, Guy's and St Thomas' NHS Foundation Trust, London, UK; 4 North East Thames Regional Genetics Service, Great Ormond Street Hospital, London, UK; 5 Centre for Cancer Genetic Epidemiology, Department of Public Health and Primary Care, University of Cambridge, Cambridge, UK; 6 Department of Clinical Genetics, Royal Devon and Exeter NHS Foundation Trust, Exeter, UK; 7 Northern Ireland Regional Genetics Centre, Belfast City Hospital, Belfast, UK; 8 Manchester Centre for Genomic Medicine, Manchester University NHS Foundation Trust, Manchester, UK; 9 Division of Evolution and Genomic Sciences, School of Biological Sciences, Faculty of Biology Medicine and Health, The University of Manchester, Manchester, UK; 10 Lothian Menopause Service, Chalmers Sexual Health Centre, Edinburgh, UK; 11 Department of Clinical Genetics, Leeds Teaching Hospitals NHS Trust, Leeds, UK; 12 Wolfson Institute of Population Health, Barts CRUK Cancer Centre, Queen Mary University of London, London, UK; 13 Department of Health Services Research and Policy, London School of Hygiene & Tropical Medicine, London, UK; 14 Department of Gynaecological Oncology, Barts Health NHS Trust, London, UK; 15 All Wales Medical Genomics Services, University Hospital of Wales, Cardiff, UK; 16 South East Scotland Clinical Genetics Service, Western General Hospital, Edinburgh, UK; 17 West Midlands Regional Genetics Service, Birmingham Women's Hospital, Birmingham, UK; 18 Department of Gynaecological Oncology, University College London Hospitals NHS Foundation Trust, London, UK; 19 Manchester Centre for Genomic Medicine, Central Manchester NHS Foundation Trust, Manchester, UK; 20 Cancer Sciences, Faculty of Medicine, University of Southampton, Southampton, UK; 21 Department of Medical Genetics, University of Cambridge, National Institute for Health Research Cambridge Biomedical Research Centre, Cambridge, UK

**Keywords:** Genetic Carrier Screening, Gynecology, Clinical Decision-Making, Delivery of Health Care, Genetic Predisposition to Disease

## Abstract

Germline pathogenic variants (GPVs) in the cancer predisposition genes *BRCA1*, *BRCA2*, *MLH1*, *MSH2*, *MSH6*, *BRIP1*, *PALB2*, *RAD51D* and *RAD51C* are identified in approximately 15% of patients with ovarian cancer (OC). While there are clear guidelines around clinical management of cancer risk in patients with GPV in *BRCA1*, *BRCA2*, *MLH1*, *MSH2* and *MSH6*, there are few guidelines on how to manage the more moderate OC risk in patients with GPV in *BRIP1*, *PALB2*, *RAD51D* and *RAD51C*, with clinical questions about appropriateness and timing of risk-reducing gynaecological surgery. Furthermore, while recognition of *RAD51C* and R*AD51D* as OC predisposition genes has been established for several years, an association with breast cancer (BC) has only more recently been described and clinical management of this risk has been unclear. With expansion of genetic testing of these genes to all patients with non-mucinous OC, new data on BC risk and improved estimates of OC risk, the UK Cancer Genetics Group and CanGene-CanVar project convened a 2-day meeting to reach a national consensus on clinical management of *BRIP1*, *PALB2*, *RAD51D* and *RAD51C* carriers in clinical practice. In this paper, we present a summary of the processes used to reach and agree on a consensus, as well as the key recommendations from the meeting.

## Background

Tubo-ovarian cancer (OC) is the sixth most common cancer in women in the UK with over 7500 new diagnoses per year.^1^ The risk of OC in first-degree relatives of patients with OC has been estimated to be threefold greater compared with the population risk. Most of this excess familial risk is due to germline pathogenic variants (GPVs) in the cancer predisposition genes *BRCA1* and *BRCA2*, which are associated with high lifetime risks of breast cancer (BC) and OC.^2^ However, other genes which have lower lifetime risks of OC have been identified. These include *MLH1*, *MSH2*, *MSH6*, *BRIP1*, *PALB2*, *RAD51D* and *RAD51C* as well as common, low-risk OC genetic susceptibility variants identified through genome-wide association studies.^3 4^

While guidelines around clinical management of cancer risk in patients with GPV in *BRCA1* and *BRCA2* 5 6 and the mismatch repair genes; *MLH1, MSH2, MSH6* 7 have been published, there are few guidelines on how to manage patients with GPV in genes associated with more moderate risks of OC: *BRIP1*, *PALB2*, *RAD51D* and *RAD51C*.

Population-based studies of genetic testing in patients with BC or OC suggest that GPV in these genes are present in a smaller but clinically important number of patients compared with GPV in *BRCA1* and *BRCA2*. Detection rates in a large population-based study of patients with BC were 274/47 522 (0.58%) for *PALB2,* 54/47 522 (0.11%) for *RAD51C,* 51/47 522 (0.11%) for *RAD51D* and 86/47 522 (0.18%) for *BRIP1*, compared with 515/48 826 (1.05%) for *BRCA1* and 754/48 826 (1.54%) for *BRCA2*.^8^ For a population-based series of patients with OC undergoing genetic testing in two states in the USA, detection rates were 0.4% (95% CI 0.11% to 1.0%), 0.58% (95% CI 0.19% to 1.3%), 0.48% (95% CI 0.13% to 1.2%) and 0.92% (95% CI 0.4% to 1.8%) for *PALB2*, *RAD51C*, *RAD51D* and *BRIP1*, compared with 8.7% *(*95% CI 7.5% to 10.1%) and 5.8% *(*5% CI 4.7% to 6.9%) for *BRCA1* and *BRCA2*.^9^ In a smaller series of 303 patients with high-grade non-mucinous OC tested through the North East London Cancer Network, the prevalence of *RAD51C*, *RAD51D* and *BRIP1* carriers was slightly higher: 0.7%, 1.0% and 0.7% for *RAD51C*, *RAD51D* and *BRIP1*, respectively, but still lower than the prevalence of *BRCA1* and *BRCA2* carriers (11% and 4.68%), respectively.^10^

There are controversies about both the appropriateness and timing of risk-reducing gynaecological surgery and also BC risk associated with GPV in these genes. International guidelines for management of individuals with *PALB2* GPV have recently been published,^11^ and *PALB2* carriers in the UK undergo breast screening via the National Health Service (NHS) Very High Risk (VHR) Breast Screening programme.^12^ While some guidelines have addressed management of OC in individuals with *RAD51C*, *RAD51D* or *BRIP1* GPV,^13^ there are no UK guidelines available for the management of all cancer risk in this patient group.

A previous UK Cancer Genetics Group (UKCGG) Consensus meeting held in 2018 agreed that *PALB2* should be included on a BC predisposition gene panel and *RAD51C*, *RAD51D* and *BRIP1* on an OC predisposition gene panel.^14^ The national genomic test directory (NHS England) was first published in August 2020 and included *PALB2* in the panel for inherited BC and OC (R208) and *RAD51C*, *RAD51D* and *BRIP1* in the inherited familial OC panel (R207). The test directory is updated each year with input from the clinical and scientific community incorporating changes to both eligibility criteria and genes on a panel (https://www.england.nhs.uk/wp-content/uploads/2018/08/Rare-and-inherited-disease-eligibility-criteria-version-3.1-August-2022.pdf). Similar panel testing is available in Wales, Scotland and Northern Ireland. Recently, evidence substantiating the role of *RAD51C* and *RAD51D* in BC predisposition was published,^8^ which suggests further amendments to the test directory may be required (see table 1 for a summary of studies assessing BC risk for *RAD51C* and *RAD51D*).

**Table 1:** Summary of key studies assessing association of BC risk in *RAD51C* and *RAD51D* GPV carriers (modified and updated from Yang *et al* 16)

Study	Cases	Controls	RR (95% CI)	
			*RAD51C*	*RAD51D*
Dorling *et al* 8	48 826 population based	50 703	OR 1.93 (1.20 to 3.11)	1.8 (1.11 to 2.93)
Hu *et al* 18	32 247 population based	32 544	1.20 (0.75 to 1.93)*	1.72 (0.88 to 3.51)*
Yang *et al* 16	6178 families, 125 with *RAD51C* GPV, and 6690 families, 60 with *RAD51D* GPV	–	1.99 (1.39 to 2.85)	1.83 (1.24 to 2.72)
Li *et al* 76	3080 with BC/EOC(Epithelial ovarian cancer)	4840	8.7 (1.9 to 80.5)	Not applicable
Susynska *et al* 77	Meta-analysis of published estimates	–	1.13 (0.88 to 1.44)	1.25 (0.9 to 1.75)
Castera *et al* 78	5131 with family history (FH) of BC/EOC	571	1.92 (0.71 to 3.85)	2.42 (0.36 to 7.39)
Huake *et al* 79	5589 eligible for mutation screening	ExAC (−27K)	OR: 1.29 to 5.91	3.04 (0.99 to 9.30)
Couch *et al* 80	38 326 eligible for mutation screening	ExAC (−27K)	0.78 (0.47 to 1.37)	3.07 (1.21 to 7.88)
Slavin *et al* 81	2135 with BC/EOC FH	ExAC (−27K)	0.39 (0.02 to 2.41)	8.33 (2.2 to 30.5)
Loveday *et al* 82	1132 families with BC/EOC FH	–	0.91 (0.45 to 1.86)	NA
Loveday *et al* 17	911 families with BC/EOC FH	–	NA	1.32 (0.58 to 2.96)

*When assessing ER-negative BC cases (n=3805), the risk association was stronger *RAD51C* 2.19 (0.97–4.49) and *RAD51D* 3.93 (1.40–10.29).

BC, breast cancer; GPV, germline pathogenic variant; NA, not applicable.

On the morning of Thursday, 30 September and the morning of Friday, 1 October 2021, a virtual consensus meeting was hosted as a collaboration between CanGene-CanVar (Cancer Research UK-funded catalyst project) and the UKCGG. The aim of the meeting was to develop guidelines around clinical management of patients with GPV in moderate-risk OC genes *PALB2*, *RAD51C*, *RAD51D* and *BRIP1* (henceforth referred to as carriers) to clarify surveillance and risk-reducing options.

## Consensus meeting

The meeting was held virtually via Zoom and was moderated by HH, FL, AK and MT on behalf of CanGene-CanVar and UKCGG. There were 65 attendees: clinical geneticists (31), genetic counsellors (9), gynaecologists (10), menopause specialists (1), clinical nurse specialists (1), breast surgeons and oncologists (5), radiologists (2), clinical scientists (1), primary care (1) and patient representatives (4). There was representation from the four devolved nations of the UK. The meeting also included commissioners (individuals involved in funding decisions at both NHSE (National Health Service England) and the devolved nations), although they did not take part in clinical discussions.

Prior to the meeting, a review of relevant literature concerning GPV in *PALB2*, *RAD51C*, *RAD51D* and *BRIP1* was undertaken, and a background document circulated summarising published evidence for associated cancers, cancer risks, surveillance and risk-reducing surgery (background document available via https://www.ukcgg.org/information-education/ukcgg-consensus-meetings/). Participants were also asked to complete a survey (via SurveyMonkey) prior to the meeting, assessing opinions on genetic testing for GPV in moderate-risk OC genes, surveillance that should be offered to carriers and options around risk-reducing surgery.

The two sessions of the meeting were divided into consideration of BC risks and management on the first morning and OC risks and management on the second morning. At the start of each session, the results from the premeeting survey were presented followed by short lectures covering risk assessment, surveillance and risk-reducing surgery by expert speakers. Following these lectures, open discussions were held around specific statements (as discussed further) to inform the clinical recommendations. Voting on each statement was undertaken using Slido, which allows real-time voting online. Statements were displayed and five options for answers from ‘strongly agree’ to ‘strongly disagree’ were proposed. Within this process, it is important to set a consensus level at the beginning.^15^ A consensus was taken as 80% of respondents agreeing (voting agree and strongly agree). Alternative statements were debated until consensus was reached. The numbers of participants voting for each statement varied, depending on the expertise of the attendees. Notes were taken throughout the meeting and a draft document of the meeting outcomes was written and edited by FL and HH and then circulated via the core group for further input and agreement.

### Question 1: should *RAD51C* and *RAD51D* be included on a BC predisposition panel?

GPV in both *RAD51C* and *RAD51D* has been identified in BC and OC families.^16 17^ For both genes, the association appears to be strongest with triple negative or ER (estrogen receptor) -negative BC.^8 18^ The largest study to date^16^ analysed data from 125 families with GPV in *RAD51C* and 60 families with *RAD51D*. This reported a relative risk (RR) of BC of 1.99 (95% CI 1.39 to 2.85) and 1.83 (95% CI 1.24 to 2.72), respectively. A large case–control analysis of 113 000 women via the Breast Cancer Association Consortium (BCAC)^8^ demonstrated similar levels of BC risk with an OR of 1.93 (95% CI 1.2 to 3.11) for *RAD51C* and 1.8 (95% CI 1.11 to 2.93) for *RAD51D*. These risks are associated with truncating GPV in these genes. An association was not demonstrated for missense variants in either *RAD51C* (OR=0.9, 95% CI 0.76 to 1.14; p=0.49) or *RAD51D* (OR=1.05, 95% CI 0.86 to 1.27; p=0.64).^8^

The cumulative lifetime risk of BC associated with truncating GPV in these genes is approximately 20% for both genes.^8 16^ However, lifetime BC risk can be significantly modified by family history with a risk as high as 44%–46% for carriers, with two first-degree relatives diagnosed with BC.^16^

- **Poll statement**: *RAD51C* and *RAD51D* should be included on a BC predisposition panel.
- **Poll results**: 33% strongly agree; 67% agree (100% consensus, n=48).
- **Recommendation**: consensus reached to include *RAD51C* and *RAD51D* on a BC predisposition panel.

### Question 2: should *BRIP1* be included on a BC predisposition panel?

In 2006 *BRIP1* was reported as a low-penetrance BC gene.^19^ However, subsequent larger and more comprehensive studies have suggested that there is not a significant association with BC predisposition.^18 20 21^ The recent BCAC study did not identify an association between truncating variants in *BRIP1* and BC risk (OR=1.11, 95% CI 0.80 to 1.53; p=0.54),^8^ and it is now widely considered that *BRIP1* is not a BC predisposition gene.

- **Poll statement**: *BRIP1* should not be included on a BC predisposition panel.
- **Poll results**: 36% strongly agree; 58% agree (94% consensus, n=50).
- **Recommendation**: consensus reached that *BRIP1* should not be included on a BC predisposition panel.

### Question 3: should the genes on a germline BC predisposition panel be the same whether the test is requested from mainstream specialty or clinical genetics?

Traditionally, diagnostic genetic testing has taken place within regional genetic services with the likelihood of identification of a GPV in a cancer predisposition gene calculated based on the family history of cancer. The main driver of testing was to facilitate predictive testing and subsequent surveillance and risk reduction strategies for unaffected family members. However, it is known that a high proportion *BRCA1* and *BRCA2* carriers do not have a significant family history of BC or OC.^22^ More recent developments in personalised cancer management for individuals with GPV have resulted in lowered thresholds for germline testing with increasing numbers of individuals now eligible. As a result, mainstreaming testing (genetic testing through a non-genetics specialty at the time of new cancer diagnosis) has been widely adopted. Predictive genetic testing in an unaffected family member for a known familial GPV in a cancer predisposition gene is undertaken through Clinical Genetics, which can provide pretest counselling and assessment of residual risk based on family history and other risk factors if an individual has not inherited a familial variant.

There have been a number of studies assessing mainstreaming pathways both from the UK^23 24^ and internationally.^25 26^ While there have been some initial concerns from non-genetic specialists around offering genomic testing,^27^ many studies have found that the mainstreaming of testing is acceptable to both patients and healthcare professionals.^10 28–31^ The group considered whether the addition of lower-risk genes to a BC predisposition panel would be of concern to mainstreaming clinicians due to unfamiliarity or uncertainty about associated cancer risks. This was weighed against the practicality of having multiple different panels, along with the consideration that all patients identified with a GPV would be referred into a genetics service for detailed discussions regarding cancer risk and surveillance/risk-reducing strategies alongside discussion of cascade testing for other at-risk family members.

- **Poll statement**: Genes on a BC predisposition panel should be the same whether requested from mainstream specialty or clinical genetics.
- **Poll results**: 24% strongly agree; 67% agree (91% consensus, n=54).
- **Recommendation**: consensus reached that genes on a BC predisposition panel should be the same, whether requested from mainstream specialty or clinical genetics.

### Question 4: what BC surveillance should be offered to patients with a GPV in *RAD51C* or *RAD51D*?

While Yang *et al* 16 estimated lifetime risks of BC of 21% and 20% for *RAD51C* and *RAD51D* carriers, respectively, these risks apply for female carriers without a significant family history. Their study also demonstrated that family history may modify this risk. With two first-degree relatives affected with BC, these lifetime risks increase to 44% and 40%, respectively.^17^ Similarly, the BCAC study suggested higher OR estimates when the comparison was made between cases with BC family history and controls. One of the more commonly used risk algorithms, CanRisk (www.canrisk.org), incorporates both family history and carrier status into individual risk assessments, and this or similar models should be used to provide an individualised risk assessment. While clinical judgement can also be used to assess the extent of family history, CanRisk provides a more detailed risk assessment and estimated 5-year, 10-year and lifetime risks, which are helpful in counselling patients and in shared decision making. The CanRisk tool can also include questionnaire-based factors (eg, hormonal and lifestyle factors), polygenic risk scores and mammographic density, although the latter two are not currently routinely assessed in standard clinical practice.

- **Poll statement**: Breast surveillance for *RAD51C* and *RAD51D* carriers should be based on an individual risk assessment.
- **Poll results**: 40% strongly agree; 60% agree (100% consensus, n=53).
- **Recommendation**: consensus reached that recommendations for breast surveillance in carriers of GPVs in *RAD51C* and *RAD51D* should be based on an individual risk assessment.

### Question 5: what breast surveillance should be offered to *RAD51C* and *RAD51D* carriers with a lifetime BC risk of 17%–30% (National institute for Health and Care Excellence (NICE) moderate-risk category)? and question 6: what breast surveillance should be offered to *RAD51C* and *RAD51D* carriers with a lifetime BC risk of >30% but <40% (NICE high-risk category)

NICE guidelines on familial BC (CG164)^6^ stratify the level of lifetime BC risk at which mammography should be offered outside of the NHS Population Breast Screening Programme (NHSBSP). Individuals are classified as having a moderate lifetime risk of BC (as opposed to an average risk) when the risk is 17%–30% or the 10-year risk is 3%–8% aged 40–50 years. The NICE guidelines for patients at moderate risk suggest annual mammography between the ages of 40 years and 49 years followed by entry into the NHS Breast Screening Programme (mammography every 3 years from age 50). These guidelines also suggest that patients with a lifetime risk of BC between 30% and 40% (10-year risk of 8%–12% aged 40–50 years) (high risk) should undergo annual mammography until the age of 59 years and then revert to population screening.

As previously mentioned, BC risk of *RAD51C* and *RAD51D* carriers can be modified by family history and other BC risk factors. Based on lifetime risk calculations undertaken in the CanRisk web tool which incorporates BOADICEA V.6 (www.canrisk.org) and considering the multifactorial model (including lifestyle/hormonal risk factors, mammographic density and polygenic risk scores), carriers can be classified into different risk categories. For an ‘average’ 20-year-old woman with a *RAD51C* GPV (without considering cancer family history), based on the multifactorial model, approximately 38% of carriers would fall into a population risk category; 43% of carriers would fall into a moderate-risk category; and 19% would fall into a high-risk category. However, for a 20-year-old *RAD51C* carrier and a mother affected with BC at age 50, based on the multifactorial model, 15% of carriers would fall into a population risk category; 42% of carriers would fall into a moderate-risk category; and 43% would fall into a high-risk category.^32^

It was noted that, in some areas of the UK, despite NICE guidelines, there remains patchy provision of moderate-risk breast screening^33^ and that access to this needs to be improved, as well as more standardised quality of reporting, as this screening currently lies outside the NHSBSP.

It was recognised that, at present, risk assessment is based predominantly on family history, but that other factors such as mammographic density and polygenic risk score could also modify risk and consequently recommendations for surveillance. The group commented that future work should focus on developing and implementing new clinical pathways that incorporate these additional risk factors.

- **Poll statement**: *RAD51C* and *RAD51D* carriers with a lifetime BC risk of 17%–30% should be offered moderate-risk surveillance according to NICE guidelines.
- **Poll results**: 27% strongly agree; 71% agree (98% consensus, n=52).
- **Recommendation**: consensus reached that *RAD51C* and *RAD51D* carriers with a lifetime BC risk of 17%–30% should be offered moderate-risk surveillance: annual mammograms 40–49 years then NHSBSP.
- **Poll statement**: *RAD51C* and *RAD51D* carriers with a lifetime BC risk of >30% but <40% should be offered high-risk surveillance according to NICE guidelines.
- **Poll results**: 20% strongly agree; 65% agree (85% consensus, n=49).
- **Recommendations**: consensus reached that *RAD51C* and *RAD51D* carriers with a lifetime BC risk of >30% but <40% should be offered high-risk surveillance annual mammograms 40–59 years then NHSBSP.

### Question 7: what breast surveillance should be offered to *RAD51C* and *RAD51D* carriers with a lifetime BC risk of 40% or greater?

The NHS VHR screening programme offers a combination of annual mammography and MRI screening to patients at VHR of BC between the ages of 25–30 years and 70 years.^12^ This is defined as ‘women with a lifetime risk of 40% or greater due to a specific genetic abnormality in the woman or her family’. To access the VHR screening programme, an individualised risk assessment using an NHS endorsed computer risk modelling software program such as CanRisk needs to be undertaken to demonstrate that 10-year BC risks are greater than 8% between the ages of 25 years and 29 years, 8% between the ages of 30 years and 39 years, or 12% between the ages of 40 years and 49 years.

While most *RAD51C* and *RAD51D* carriers are unlikely to reach this level of BC risk, a small number of patients may reach this level of risk based on the strength of their family history and/or other modifying factors.^16^

- Poll statement: *RAD51C* and *RAD51D* carriers with a lifetime BC risk of ≥40% should be referred to the VHR Breast Screening programme.
- **Poll results**: 23% strongly agree; 75% agree (98% consensus, n=48).
- **Recommendations**: consensus reached that *RAD51C* and *RAD51D* carriers with a lifetime BC risk of ≥40% should be referred to the NHS VHR Breast Screening programme at the appropriate age following an individualised risk assessment.

### Question 8: when should risk-reducing mastectomy (RRM) be discussed with *RAD51C* and *RAD51D* carriers?

NICE guidelines include RRM as part of the pathway for managing patients with a VHR of BC. There is a strong body of evidence demonstrating a greater than 90% risk reduction associated with RRM in patients with GPV in *BRCA1* and *BRCA2*.^34^ There is also emerging evidence that surgery will increase survival.^35–37^ However, no formal study of *RAD51D* or *RAD51C* carriers has been undertaken. The group discussed that as for patients with a strong family history of BC who meet a 30% or greater lifetime risk of BC, discussion of the option of RRM with a patient is appropriate. However, discussion with the patient should be based on individual circumstance and shared decision making. Given the lack of studies for *RAD51C* and *RAD51D* carriers, detailed counselling with patients should include, but not be restricted to, individualised cancer risk assessment, personal circumstance and preferences of the counsellee. Non-genetic risk factors such as dense breast tissue, hormonal/lifestyle modifiers and other pre-existing medical conditions should also be considered. Importantly, age-specific risksand 5 and 10 year risks, should be communicated to the patient to help in their decision making.

It was noted that some patients may fall into a level of risk where RRM, but not surveillance within the VHR surveillance programme, is offered (lifetime risk 30%–39% inclusive). In this situation, particular consideration should be paid to detailed discussion of age-specific risks.

- **Poll statement**: RRM should be discussed with *RAD51C* and *RAD51D* carriers with a lifetime BC risk of ≥30%.
- **Poll results**: 16% strongly agree, 78% agree (94% consensus, n=50).
- **Recommendation**: consensus reached that RRM should be discussed with *RAD51C* and *RAD51D* carriers with a lifetime BC risk of ≥30%, in conjunction with an individualised risk assessment, appropriate counselling and shared decision making.

### Question 9: should OC surveillance be offered to *RAD51C*, *RAD51D*, *BRIP1* and *PALB2* carriers?

Currently, population screening for OC is not recommended in the UK due to lack of evidence of a mortality benefit.^38^ While there have been several studies assessing screening in those at increased risk of OC (carriers of GPV in *BRCA1* and *BRCA2*), these have also not demonstrated a clear utility. The UK FOCSS (UK Familial Ovarian Cancer Screening Study) reported that risk of ovarian cancer algorithm (ROCA)-based screening (screening with CA-125, interpreted using the ROCA and transvaginal sonography (TVS)) demonstrated a stage shift for patients at high risk of OC. However, it remains unknown whether this surveillance would improve survival in screened high-risk patients.^39^ The recently published Avoiding Late Diagnosis of Ovarian Cancer (ALDO) project, evaluated the utility of ROCA in *BRCA1* and *BRCA2* carriers (not available at the time of the consensus meeting), confirmed previous research that although this approach cannot prevent ovarian cancer, in women who defer RRSO, it may facilitate detection of ovarian cancer at earlier stages, resulting in less complex surgery and reduced need for pre-op chemotherapy.^40^ Recent guidelines on the management of individuals with *PALB2* GPV did not recommend OC surveillance.^11^ Studies specifically assessing surveillance in individuals with GPV in *RAD51C*, *RAD51D* or *BRIP1* have not yet been undertaken. Therefore, currently, there are no national recommendations for OC surveillance for patients at increased risk based on family history and/or genetic status, and currently, no test has been shown to detect the majority of high-grade serous OC prior to metastatic disease, either in a population or high-risk setting. General population randomised controlled trials have not shown a mortality benefit,^38^ and there is currently no national OC screening programme.

- **Poll statement**: OC surveillance should not routinely be offered to *RAD51C*, *RAD51D*, *BRIP1* and *PALB2* carriers.
- **Poll results**: 13% strongly agree; 70% agree (83% consensus, n=47).
- **Recommendation**: consensus reached that OC surveillance should not routinely be offered to *RAD51C*, *RAD51D*, *BRIP1* and *PALB2* carriers.

### Question 10: should OC surveillance be offered to *RAD51C*, *RAD51D*, *BRIP1* and *PALB2* carriers within a research study?

Given the lack of specific studies of the utility of OC surveillance in *RAD51C*, *RAD51D*, *BRIP1* and *PALB2* carriers, the group discussed whether surveillance could or should be offered within the context of a research study. While the majority of the group agreed with this approach, there was also concern that if OC surveillance in *BRCA1* and *BRCA2* carriers was supported in the future, then it would be difficult not to extend this to moderate-risk gene carriers, as OC in *RAD51C*, *RAD51D*, *BRIP1* and *PALB2* carriers is similarly, most likely to be high-grade serous OC, compared with other OC pathologies.^41^ While there is no formal analysis, it is likely that the cost per OC case detected would be higher for the moderate-risk genes compared with BRCA carriers, given the overall incidence of OC is lower. However, the absolute cost of adding *RAD51C*, *RAD51D*, *BRIP1* and *PALB2* carriers to any pre-existing high-risk programme would be low due to relatively small numbers.^41^ Overall, there was consensus that surveillance should only be offered in the context of a research study at present, but that this could be reviewed if national recommendations in the future support surveillance in a high-risk population.

- **Poll statement**: OC surveillance should only be offered to *RAD51C*, *RAD51D*, *BRIP1* and *PALB2* carriers within an ethically approved research study.
- **Poll results**: 20% strongly agree; 76% agree (96% consensus, n=47).
- **Recommendation**: consensus reached that OC surveillance should only be offered to *RAD51C*, *RAD51D*, *BRIP1* and *PALB2* carriers within the context of a research study.

### Question 11: what should the lifetime risk of OC for a *RAD51C*, *RAD51D*, *BRIP1* and *PALB2* carrier be based on for clinical discussions?

Yang *et al* 16 reported a RR of OC of 7.55 (95% CI 5.6 to 10.19) for *RAD51C* GPV and 7.6 (95% CI 5.61 to 10.3) for *RAD51D* GPV. The cumulative lifetime risks of OC (to age 80 years) are estimated to be 11% (95% CI 6 to 21%) and 13% (95% CI 7 to 23%), respectively. This risk has been shown to be modified by family history of OC, with a risk exceeding 30% for carriers with two first-degree relatives with OC. The risks for *RAD51C* and *RAD51D* carriers are largely conferred after the age of 50 years.

Data from a meta-analysis of carriers of GPV in *BRIP1* 42 calculated an OR of 4.94 (95% CI 4.07 to 6.00) for OC. A further study^43^ calculated a cumulative risk of OC to the age of 80 years of 5.8% (95% CI 3.6 to 9.1%). This study gave a larger range of OR for risks which may be indicative of the influence of family history of OC on risk.

Presence of a GPV in *PALB2* was demonstrated in a study of 852 carriers^44^ to give an RR of OC of 2.91 (95% CI 1.4 to 6.04). Age-specific risks were then calculated with the lifetime risk to 80 years being quoted as 5% (95% CI 2% to 10%). A further study of risks estimated the cumulative lifetime risk to age 80 years to be 3.2% (95% CI 1.8% to 5.7%).^45^ As with *RAD51C* and *RAD51D*, the risk of OC associated with GPV in *PALB2* appears to be modified by family history of OC. For example, female carriers with a mother and sister with OC diagnosed at 50 years have a lifetime risk of 16% (95% CI 8% to 28%).^44^

In addition to family history, it is recognised that other hormonal and lifestyle factors can modify OC risk, including use of oral contraception, hormonal replacement therapy (HRT), parity, body mass index, tubal ligation and endometriosis. All these factors can be incorporated into the CanRisk model alongside family history to provide an individualised risk assessment. In addition, polygenic risk scores may also modify risk in either direction but at present are not available in routine clinical practice.

- **Poll statement**: Lifetime risk of OC for a *RAD51C*, *RAD51D*, *BRIP1* and *PALB2* carrier should be based on an individualised risk assessment taking family history into consideration.
- **Poll results**: 35% strongly agree; 63% agree (98% consensus, n=51).
- **Recommendation**: consensus reached that lifetime risk of OC of a *RAD51C*, *RAD51D*, *BRIP1* and *PALB2* carrier should be based on an individualised risk assessment taking family history into consideration.

### Question 12: when discussing risk-reducing bilateral salpingo-oophorectomy (RRSO) with a patient, what risks should be considered?

The risks of OC for *RAD51D* and *RAD51D* carriers are largely conferred after the age of 50 years. The highest 10-year risk with GPV in *RAD51C* is between the ages of 50 and 60 with the highest risks between 50 and 70 years in *RAD51D* carriers (Yang, personal communication, 2021). These risks as previously described vary with family history.^16^ To discuss RRSO, both lifetime and 5-10 year OC risks should be considered, balancing risks versus benefits of surgery, with particular consideration to the average age of menopause in the population and individual menopause status of the patient. Other issues such as fertility and the impact of premature menopause may also affect timing of surgery and patient decision making.

For *BRIP1* carriers, the majority of OC risk also occurs after the age of 50 years. The average age of diagnosis for a *BRIP1* carrier was at age 63.8 years compared with 58 years in non-carriers.^43^ This was replicated in a study assessing 222 patients with OC and *BRIP1* GPV.^46^ In this study, 90% of cases occurred after the age of 50 years with a median age of 65 years. A recent study suggested that the *BRIP1* variant c.1045G>C is a higher-risk allele,^47^ although this still demonstrated a mean age of diagnosis of 62.5 years.

Yang *et al*,^44^ in a study of 852 female *PALB2* carriers, demonstrated that the majority of OC risk is over the age of 50 years, with an estimated cumulative risk below this age of less than 1%. This was supported by a further study^45^ with a cumulative risk of OC of less than 1% under the age of 50.

- **Poll statement**: Discussion of RRSO should consider both lifetime risk and risks of 5–10 years.
- **Poll results**: 23% strongly agree; 68% agree (91% consensus, n=53).
- **Recommendation**: consensus reached that discussion of RRSO should consider both lifetime risk and 5-10 year risks

### Question 13: what discussions should take place when considering premenopausal RRSO?

It is recognised that in young women undergoing surgical oophorectomy, there is an impact on both morbidity and mortality.^48^ The sequelae can include vasomotor symptoms, decrease in sexual function, osteoporosis, increased risk of cardiovascular disease, depression, anxiety, dementia and cognitive decline, and multimorbidity.^49–53^ While HRT is routinely recommended and a number of these outcomes are attenuated by the use of HRT, not all of the sequelae are fully mitigated by this.^51 53–62^ Other issues that need to be considered include fertility. RRSO is only recommended once childbearing is complete.

Studies have demonstrated need for information about the postsurgical effects of RRSO.^63 64^ The group considered the need for detailed discussions with patients to ensure they are aware of the potential side effects so that these can be balanced with individualised discussion of risk.

- **Poll statement**: Discussion of premenopausal RRSO should include a full detailed discussion of OC risk versus potential sequelae of early menopause.
- **Poll results**: 72% strongly agree, 28% agree (100 consensus, n=53).
- **Recommendation**: consensus reached that discussion of premenopausal RRSO should include a full detailed discussion of OC risk versus potential sequelae of early menopause.

### Question 14: at what level of risk should RRSO be offered?

Historically, in UK practice, a lifetime OC risk of 10% has been used as the threshold of risk for discussion of RRSO. However, prior to comprehensive risk assessment models for OC, both with and without a recognised causative GPV in a family, calculation of individualised risk has been complex. As a result, most typically, discussion of RRSO has been based on either a genetic diagnosis in a family or clinically based criteria.For example, two or more cases of OC in a family. Recent studies in the UK^65 66^ have demonstrated the cost-effectiveness of RRSO above a threshold of 4%–5% lifetime risk. These studies suggest that patients with a lifetime risk above this threshold should be offered the opportunity to discuss RRSO. However, while surgery will decrease OC risk, there are potentially long-term sequelae associated with surgical oophorectomy, as described previously.

Considering the aforementioned, the risk threshold for offering RRSO was discussed. The group considered that counselling of patients should include an individualised risk assessment with discussion of both lifetime risk and 5-10 year risks (see question 12) that takes genetic test results and family history as a minimum into the risk assessment. Discussion should also include counselling on the possible sequelae of an early menopause (see question 13).

- **Poll question**: At what threshold of risk should RRSO be offered? (options: <5%, 5%–10% and >10%)
- **Poll results**: 4% <5% lifetime risk, 79% 5%–10% lifetime risk, 13% over 10% lifetime risk, 4% uncertain (n=53).
- **Recommendation**: consensus reached that RRSO should be discussed at a lifetime risk of OC of ≥5%.

### Question 15: at what age should RRSO be considered for *RAD51C* carriers?

Yang *et al* demonstrated^16^ that the cumulative risk of OC up until 50 years was 1% (95% CI 0.6% to 2.0%) with a risk to 80 years of 11% (95% CI 6% to 21%). CanRisk data presented in the meeting by AA demonstrated that the average OC risk for a *RAD51C* carrier is ~11% to age 80 years, with only ~5% carriers falling below a lifetime risk of 5% based on the multifactorial OC risk model. When considering risk to age 50 years, the average risk is 1.1% and based on the multifactorial model, 99.6% of carriers fall below a 3% risk before age 50 years^32 67^ (table 2A). The risk-to-benefit ratio for surgery therefore changes over the age of 50 years. However, risk can also be modified by family history, and the risk classifications presented earlier, based on the multifactorial model, will also be family history specific (table 2B). Therefore, the group considered that an individualised risk assessment, as well as assessment of menopausal status, should be undertaken for all *RAD51C* carriers. The initial poll statement ‘For *RAD51C* carriers RRSO should only rarely be considered <50 years and should include individualised risk assessment and shared decision making’ was reworded following detailed group discussion to reflect the importance of these considerations to ‘For *RAD51C* carriers with a 5% or greater lifetime risk, RRSO should be considered at 50. It can be considered in patients younger than 50 following individualised risk assessment including assessment of menopausal symptoms and shared decision making’.

**Table 2:** OC risk categorisation to ages 50 and 80 years for *RAD51C*, *RAD51D*, *BRIP1* and *PALB2* GPV carriers based on the multifactorial OC model^67^ implemented in CanRisk (A Antoniou, personal communication 2022, and adapted from data in Lee *et al* 32)

Gene	Considering OC risk to age 80 years				Considering OC risk to age 50 years			
	Average risk to age 80 (%)*	% carriers in risk category†			Average risk to age 50 (%)*	% carriers in risk category†		
		<5% risk	5%–10% risk	>10% risk		<3% risk	3–5% risk	>5% risk
(A) For an unaffected GPV carrier, unselected due to family history of OC								
*RAD51D*	13	2	33	65	0.9	99.9	0.1	0
*RAD51C*	11	5	44	51	1.1	99.6	0.4	0
*BRIP1*	6	47	47	6	1	99.7	0.2	0
*PALB2*	5	62	35	3	0.8	100	0	0
(B) For a GPV carrier with a mother affected with OC, age 50								
*RAD51D*	23	0	98	1.9	95.4	4.4		0.2
*RAD51C*	20	0.1	94.6	2.3	89.2	10.1		0.8
*BRIP1*	11	5	49	2	91.6	7.9		0.5
*PALB2*	10	11	33	1.5	97.5	2.4		0.1

*Average risk for a GPV carrier, based only on the GPV.

†Based on the multifactorial risk model, including questionnaire-based/clinical risk factors and polygenic risk score.

GPV, germline pathogenic variant; OC, ovarian cancer.

- **Poll question**: For *RAD51C* carriers with a 5% or greater lifetime risk, RRSO should be considered at 50. It can be considered in carriers younger than 50 following individualised risk assessment, including assessment of menopausal symptoms and shared decision making.
- **Poll results**: 14% strongly agree; 86% agree (100% consensus, n=43).
- **Recommendation**: consensus reached that for *RAD51C* carriers with a 5% or greater lifetime risk, RRSO should be considered at 50 years. It can be considered in carriers younger than 50 years following individualised risk assessment including assessment of menopausal symptoms and shared decision making.

### Question 16: at what age should RRSO be considered for *RAD51D* carriers?

Yang *et al* estimated that the cumulative risk of OC for *RAD51D* carriers to 50 years is 0.8% (95% CI 0.5% to 2.0%) and 13% (95% CI 7% to 23%) to age 80 years. CanRisk data presented in the meeting by AA consider that the average OC risk for a *RAD51D* carrier is 13% to age 80 years. Based on the multifactorial model, 2% of *RAD51D* carriers fall below a lifetime risk of 5%. However, when considering risk to age 50 years, the average risk is 0.9%, and based on the multifactorial model, 99.9% of carriers fall below a 3% risk before age 50 years (table 2A). This risk can be influenced by family history, with risk rising with increasing number of first-degree relatives affected with OC and risk classification also dependent on cancer family history (table 2B). Like the discussions for *RAD51C* carriers, the group felt that the age at which to consider RRSO should reflect both an individualised risk assessment and also menopausal status, and rewording of the poll question for voting was undertaken in real time during the meeting to reflect this.

- **Poll question**: For *RAD51D* carriers with a 5% or greater lifetime risk, RRSO should be considered at 50. It can be considered in carriers younger than 50 following individualised risk assessment including assessment of menopausal symptoms and shared decision making.
- **Poll results:** 14% strongly agree; 86% agree (100% consensus, n=44).
- **Recommendations**: consensus reached that for *RAD51D* carriers with a 5% or greater lifetime risk, RRSO should be considered at 50 years. It can be considered in carriers younger than 50 years following individualised risk assessment, including assessment of menopausal symptoms and shared decision making.

### Question 17: at what age should RRSO be considered for *BRIP1* carriers?

The cumulative OC risk associated with pathogenic variants in *BRIP1* is 5.8% (95% CI 3.6% to 9.1%). It would appear that the risk is highest after the age of 50 years, with the average diagnosis being at 63 years.^43^ A further study of *BRIP1* carriers demonstrated that 90% developed OC after the age of 50, with a median age of diagnosis of 65 years.^46^ CanRisk data presented in the meeting model the average OC risk for a *BRIP1* carrier to be ~6% to age 80 years, and based on the multifactorial model, 47% carriers fall below a lifetime risk of 5%. When considering risk to age 50 years, the average risk is 1.0%, with 99.7% of carriers falling below a 3% risk before age 50 years when using the multifactorial model (table 2A). Again, reflecting the previous discussions for *RAD51C* and *RAD51D* carriers, the group felt that the age at which to consider RRSO for *BRIP1* carriers should include both an individualised risk assessment and also menopausal status, and rewording of the poll question for voting was undertaken in real time during the meeting to reflect this. It was also noted that unlike *RAD51C* and *RAD51D*, many *BRIP1* carriers, in the absence of OC family history, may not reach a lifetime OC risk of 5%, and calculation of an individualised risk assessment was therefore fundamental to patient discussions (table 2A, B).

- **Poll question**: For *BRIP1* carriers with a 5% or greater lifetime risk, RRSO should be considered at 50. It can be considered in carriers younger than 50 following individualised risk assessment, including assessment of menopausal symptoms and shared decision making.
- **Poll results**: 9% strongly agree; 91% agree (100% consensus, n=43).
- **Recommendation**: consensus reached that for *BRIP1* carriers with a 5% or greater lifetime risk, RRSO should be considered at 50 years. It can be considered in carriers younger than 50 years following individualised risk assessment, including assessment of menopausal symptoms and shared decision making.

### Question 18: at what age should RRSO be considered for *PALB2* carriers?

A study of 852 *PALB2* carriers calculated age-specific OC risks. It was estimated that there is 0.6% risk (95% CI 0.3% to 1.0%) to 50 years and 5% (95% CI 2% to 10%) to age 80 years.^44^ A further study suggested the risk is 3.2% until 80 years.^45^ Family history can modify this risk. Given low cumulative risk under the age of 50 years, recent guidelines from the American College of Medical Genetics and Genomics suggested that RRSO be discussed only from 50 years onwards.^11^ CanRisk data presented in the meeting model the average OC risk for a *PALB2* carrier to be ~5% to age 80 years, with many *PALB2* (62%) carriers falling below a lifetime risk of 5%, based on the multifactorial model and absence of OC family history (table 2A). When considering risk to age 50 years, the average risk is 0.8%, with all carriers (without family history of OC) falling below a 3% risk before age 50 years (table 2A). The group felt, in general, more cautious about recommendations for RRSO for *PALB2* carriers, compared with *RAD51C and RAD51D*. The importance of individualised risk assessment was emphasised, given that only a small number of *PALB2* carriers, without OC family history are likely to reach a lifetime OC risk of 5%, but if there is a family history of OC, a larger proportion of carriers will reach a lifetime risk of 5% (table 2A, B).

- **Poll question**: For *PALB2* carriers with a 5% or greater lifetime risk, RRSO should be considered at 50. It can be considered in carriers younger than 50 following individualised risk assessment, including assessment of menopausal symptoms and shared decision making.
- **Poll results**: 12% strongly agree; 86% agree (98% consensus, n=42).
- **Recommendation**: consensus reached that for *PALB2* carriers with a 5% or greater lifetime risk, RRSO should be considered at 50 years. It can be considered in carriers younger than 50 years following individualised risk assessment, including assessment of menopausal symptoms and shared decision making.

### Question 19: should the option of risk-reducing early salpingectomy with delayed oophorectomy be considered for *RAD51C*, *RAD51D*, *BRIP1 and PALB2* carriers?

There is now evidence that OC can arise from the fallopian tubes and that removal of the fallopian tubes may therefore decrease the risk of OC.^68–70^ This raises the possibility of using salpingectomy with delayed oophorectomy to decrease OC risk while delaying surgical menopause in carriers at increased risk of OC.

A recent study from the Netherlands assessed quality of life after risk-reducing salpingectomy versus RRSO in 577 *BRCA1* and *BRCA2* carriers. This demonstrated a better quality of life after risk-reducing salpingectomy irrespective of HRT.^71^ Within the UK, the PROTECTOR study is evaluating the option of risk-reducing early salpingectomy with delayed oophorectomy in patients at high risk of OC. *BRCA1*, *BRCA2*, *PALB2*, *BRIP1*, *RAD51C* and *RAD51D* carriers are eligible for this study.^72^

While there are several ongoing studies about the acceptability of this approach, there are still no long-term data on outcomes, in particular around OC diagnoses in these cohorts. Recent reviews by Boerner *et al* and Gaba *et al* suggested that this surgery should be offered only in the context of a clinical trial.^72 73^

- **Poll question**: Risk-reducing early salpingectomy with delayed oophorectomy should be offered only to *RAD51C*, *RAD51D*, *BRIP1* and *PALB2* carriers within the context of a research study.
- **Poll results**: 30% strongly agree; 63% agree (93% consensus, n=40).
- **Recommendation**: consensus reached that risk-reducing early salpingectomy with delayed oophorectomy should currently be offered only to *RAD51C*, *RAD51D*, *BRIP1* and *PALB2* carriers within the context of a research study until further data are available.

### Question 20: is there a role for OC surveillance for *RAD51C*, *RAD51D*, *BRIP1* and *PALB2* carriers who have opted not to pursue risk-reducing surgery?

The current evidence for surveillance for OC suggests that it is ineffective in the general population. The long-term follow-up results for the UKTOCS (UK Collaborative Trial of Ovarian Cancer Screening) have recently been published^38^ and demonstrate that surveillance does not decrease deaths from ovarian or tubal cancers. The UKFOCSS study reported that ROCA-based screening (screening with CA-125, interpreted using the ROCA, and TVS) for patients at high risk of OC demonstrated a stage shift. However, it remains unknown whether this screening would improve survival in screened high-risk patients.^39^ Previous discussion in the meeting had reached consensus that OC surveillance should only be offered to *RAD51C*, *RAD51D*, *BRIP1* and *PALB2* carriers within a research study (see question 10). The group also considered whether there was any role for surveillance for carriers who had opted not to pursue risk-reducing surgery.

- **Poll question**: OC surveillance can be considered for *RAD51C*, *RAD51D*, *BRIP1* and *PALB2* carriers who have opted not to pursue risk-reducing surgery.
- **Poll results:** 84% disagree; 2% strongly disagree (86% consensus, n=49).
- **Recommendation**: Carriers of GPVs in *RAD51C, RAD51D, BRIP1* and *PALB2 should not* be offered surveillance (outside the setting of a research study) even if they have opted not to pursue risk-reducing gynaecological surgery.

## Discussion

Clear management guidelines exist for *BRCA1* and *BRCA2* carriers with guidance around surveillance and risk-reducing surgery for BC and OC. While the contribution of GPV in *PALB2*, *BRIP1*, *RAD51C* and *RAD51D* is smaller than for *BRCA1* and *BRCA2*, the inclusion of these genes on breast and OC gene panels has resulted in the need for similar guidance around management of carriers for GPV in these genes. However, there are no guidelines setting out surveillance or encompassing all cancer risk management available in the UK. As with much of clinical genetics, clear evidence of the optimal management of affected individuals is scarce due to the rarity of the disease and requirement for very long-term follow-up studies to generate data on which to base guidelines.

A multidisciplinary workshop was therefore convened to draw on expert clinical experience. By the end of the two-session workshop, a consensus (over 80% agreement) had been obtained for a majority of recommendations for best clinical practice for carriers of GPV in *RAD51C*, *RAD51D*, *PALB2* and *BRIP1*. A summary is presented in Box 1.

Box 1 **Summary of clinical recommendations for genes discussed in consensus meeting**

**Recommendations for *RAD51C* and *RAD51D* carriers**

- *RAD51C* and *RAD51D* should be included on a BC predisposition gene panel.
- Breast surveillance for *RAD51C* and *RAD51D* carriers should be based on an individualised risk assessment.
  - Carriers with a lifetime BC risk of 17%–30% should be offered moderate-risk breast surveillance: annual mammograms 40–49 then NHSBSP.
  - Carriers with a lifetime BC risk of >30% but <40% should be offered high-risk breast surveillance annual mammograms 40–59 years then NHSBSP.
  - Carriers with a lifetime BC risk of >40% should be referred to the VHR Breast Screening programme.
- Risk reducing mastectomy can be discussed with *RAD51C* and *RAD51D* carriers with a lifetime BC risk of ≥30%, in conjunction with an individualised risk assessment, appropriate counselling and shared decision making.
- OC surveillance should not routinely be offered to *RAD51C* or *RAD51D* carriers outside a research study
- Discussion of lifetime risk of OC for *RAD51C* and *RAD51D* carriers should be based on an individualised risk assessment, considering both lifetime risk and risks of 5–10 years and taking family history into consideration.
- For *RAD51C* and *RAD51D* carriers, with a 5% or greater lifetime risk of OC, RRSO should be considered at 50 years. It can be considered in carriers younger than 50 years following individualised risk assessment including assessment of menopausal symptoms and shared decision making.
- Discussion of premenopausal RRSO should include a full detailed discussion of risk versus side effects due to an early menopause.
- Risk reducing early salpingectomy with delayed oophorectomy should only be offered to *RAD51C* and *RAD51D* carriers within a research study.

**Recommendations for *BRIP1* carriers**

- *BRIP1* should *not* be included on a BC gene predisposition panel.
- Breast surveillance for *BRIP1* carriers should be based on family history and not *BRIP1* carrier status.
- OC surveillance should not routinely be offered to *BRIP1* carriers outside a research study.
- Discussion of lifetime risk of OC for *BRIP1* carriers should be based on an individualised risk assessment, considering both lifetime risk and risks of 5–10 years and taking family history into consideration.
- For *BRIP1* carriers with a 5% or greater lifetime risk, RRSO should be considered at 50 years. It can be considered in carriers younger than 50 years following individualised risk assessment, including assessment of menopausal symptoms and shared decision making.
- Discussion of premenopausal RRSO should include a full detailed discussion of risk versus side effects due to an early menopause.
- Risk reducing early salpingectomy with delayed oophorectomy should only be offered to *BRIP1* carriers within a research study.

**Recommendations for *PALB2* carriers**

- *PALB2* should be included on a breast cancer predisposition gene panel.
- Breast surveillance for *PALB2* carriers should be based on an individualised risk assessment with carriers referred to the NHSBSP VHR screening programme at age 25–30, depending on risk.
- RRM can be discussed with *PALB2* carriers with a lifetime BC risk≥30%, in conjunction with an individualised risk assessment, appropriate counselling and shared decision making
- OC surveillance should not routinely be offered to *PALB2* carriers outside a research study.
- Discussion of lifetime risk of OC for *PALB2* carriers should be based on an individualised risk assessment, considering both lifetime risk and risks of 5–10 years and taking family history into consideration.
- For *PALB2* carriers, with a 5% or greater lifetime risk, RRSO should be considered at 50 years. It can be considered in carriers younger than 50 years following individualised risk assessment, including assessment of menopausal symptoms and shared decision making.
- Discussion of premenopausal RRSO should include a full detailed discussion of risk versus side effects due to an early menopause.
- Risk-reducing early salpingectomy with delayed oophorectomy should only be offered to *PALB2* carriers within a research study.

In summary, carriers of GPV in these genes should have a detailed discussion about their family history, individualised risk assessment and offered RRSO at the appropriate level of risk and age. *RAD51C*, *RAD51D* and *PALB2* carriers should also have an individualised risk assessment for BC and be entered into the appropriate breast screening programme.

While these guidelines suggest ‘best practice’ management, there are a number of issues which impact on the implementation of the guidelines, including issues around resources and geographical differences in the delivery of care. It is suggested that *RAD51C* or *RAD51D* carriers should have enhanced BC risk screening as defined in the NICE CG164 familial BC guidelines.^6^ However, from discussions in the workshop, it is apparent that, in line with a previous publication,^33^ provision of services for moderate-risk and high-risk breast surveillance varies around the UK. This needs to be addressed, not least in response to the NHS Long Term Plan (2019), which aims to increase the number of early cancer diagnosis by screening. Populations at increased risk of malignancy, such carriers of GPV in these moderate-risk genes, are populations for targeted screening, fulfilling criteria set by the National Screening Committee (https://www.gov.uk/government/publications/evidence-review-criteria-national-screening-programmes/criteria-for-a-targeted-screening-programme).

VHR breast screening is included within the NHSBSP for England, and there is now agreement to offer colonoscopy surveillance for individuals with Lynch syndrome within the NHS Bowel Screening programme with roll-out anticipated in 2023. One possibility is that moderate-risk and high-risk screening could be incorporated into the NHSBSP (as for VHR) so current inequities for access and reporting can be improved and standardised. However, there are issues with the funding and services offered to the devolved nations resulting in disparate provision across the UK.

The workshop has produced clear guidance around recommendations of risk levels at which to consider RRSO. However, it should be remembered that the risk estimates based on multifactorial risk models may be associated with wide CIs based on the uncertainty related to input parameters. Risk estimates are also dependent on the accuracy, quality, validity and extent of the information input into the tool. External, independent validation studies provide useful guidance on these aspects.^74^ As such, discussions with patients will require explanation of the variation in risk estimations along with detailed discussions about timing of surgery and potential sequelae. This will then facilitate shared decision making with each individual patient to optimise care as per NICE guidance 197.^75^ These sequelae may include menopausal symptoms, depending on the time of the surgery. Women should have access to discussions around HRT or alternative therapies when appropriate. However, it was highlighted in the workshop that while some centres of excellence exist, access to specialised menopausal care is variable around the UK.

Overall, with increased numbers of *RAD51C*, *RAD51D*, *BRIP1* and *PALB2* carriers likely to be identified imminently with updates to the national test directory within all four UK nations, we believe that these guidelines represent a framework for consistent and best practice based on the current evidence. It is likely that new relevant information will be published in the next 5 years, both from larger studies of carriers and results from studies addressing ovarian surveillance and risk-reducing surgery; therefore, regular review and updates will be required and discussions with patients should also include the potential for clinical recommendations to change over time, as and when new evidence becomes available.
